# Meeting Report: Threats to Human Health and Environmental Sustainability in the Pacific Basin

**DOI:** 10.1289/ehp.9620

**Published:** 2007-10-18

**Authors:** Robert G. Arnold, David O. Carpenter, Donald Kirk, David Koh, Margaret-Ann Armour, Mariano Cebrian, Luis Cifuentes, Mahmood Khwaja, Bo Ling, Irma Makalinao, César Paz-y-Miño, Genandrialine Peralta, Rajendra Prasad, Kirpal Singh, Peter Sly, Chiharu Tohyama, Alistair Woodward, Baoshan Zheng, Todd Maiden

**Affiliations:** 1 Department of Chemical and Environmental Engineering, The University of Arizona, Tucson, Arizona, USA; 2 Institute for Health and the Environment, University at Albany, State University of New York, Albany, New York, USA and Rensselaer Polytechnic Institute, Troy, New York, USA; 3 Department of Chemical Engineering and Applied Chemistry, University of Toronto, Toronto, Canada; 4 Department of Community, Occupational and Family Medicine, National University of Singapore, Kent Ridge, Republic of Singapore; 5 Department of Chemistry, University of Alberta, Edmonton, Alberta, Canada; 6 Toxicology Section, Center for Research and Advanced Studies, Instituto Politécnico Nacional, Mexico City, Mexico; 7 Department of Engineering and Industrial Systems, Universidad Católica de Chile, Santiago, Chile; 8 Sustainable Development Policy Institute, Islamabad, Pakistan; 9 Institute of Environmental Health and Engineering, Chinese Academy for Preventive Medicine, Shanghai, People’s Republic of China; 10 Department of Pharmacology and Toxicology, University of the Philippines, Manila, Republic of the Philippines; 11 Department of Molecular Genetics and Cytogenetics Human Laboratory, Catholic University of Ecuador, Quito, Ecuador; 12 National Engineering Center, University of the Philippines, Manila, Republic of the Philippines; 13 International Science and Technology Affairs, Council of Scientific and Industrial Research, New Delhi, India; 14 Jubilee University, Papua New Guinea; 15 TVW Telethon Institute for Child Health, West Birth, Australia; 16 Center for Disease Biology and Integrated Medicine, The University of Tokyo, Tokyo, Japan; 17 School of Population Health, The University of Auckland, Auckland, New Zealand; 18 State Key Laboratory of Environmental Geochemistry, Institute of Geochemistry, Guiyang, People’s Republic of China; 19 Reed Smith, Ltd., San Francisco, California, USA

**Keywords:** arsenic, electronics waste, environmental sustainability, global climate change, hazardous waste, human health, Pacific basin, persistent organic pollutants, pesticides

## Abstract

The coastal zone of the Pacific Rim is home for about one-third of the world’s population. Disproportionate growth of Far Eastern economies has produced a disproportionate share of related environmental difficulties. As the region searches for acceptable compromises between growth and environmental quality, its influence on global environmental health is certain to increase. Consequences of global environmental change such as habitat alteration, storms, and sealevel rise will be particularly acute among Pacific Rim nations. Adverse health effects from arsenic exposure in Pacific Rim nations have been used to justify drinking water standards in the United States and elsewhere. As global manufacturing in the Pacific Rim increases, the centroid of global air quality and waste management issues will shift further toward Far Eastern nations. The Eleventh International Conference of the Pacific Basin Consortium (PBC) was held in September 2005 in Honolulu, Hawaii. The purpose of the conference was to bring together individuals to discuss regional challenges to sustainable growth. The historic emphasis of the conference on hazardous wastes in relation to human health makes the PBC an ideal forum for discussing technical aspects of sustainable economic growth in the Pacific region. That role is reflected in the 2005 PBC conference themes, which included management of arsenic in potable waters, air quality, climate change, pesticides, mercury, and electronics industry waste—each with emphasis on relationships to human health. Arsenic management exemplifies the manner in which the PBC can focus interdisciplinary discussion in a single technical area. The conference program provided talks on arsenic toxicology, treatment technologies, management of arsenic-bearing residuals from water treatment, and the probable societal costs and benefits of arsenic management.

## Mission of the Pacific Basin Consortium

About 2 billion people live in nations on the Pacific Rim. These include the most populous countries in the world as well as some of the wealthiest and the poorest. As significantly, technical and industrial growth statistics suggest that Pacific Rim nations are in the midst of economic expansions that can both alter regional health risk factors and support measures to protect environmental and human health. Regional interests in this area are best served through cooperation among Pacific Rim nations, acknowledging very significant differences in culture, language, government, religion, development, and wealth.

The primary goal of the Pacific Basin Consortium for Environment and Health Sciences (PBC) is to promote multidisciplinary, multinational efforts to improve regional human and environmental health. Steps toward that goal include recognition of existing health-related problems and the new threats that will accompany rapid regional economic and industrial transitions. This was the theme of the Eleventh International Conference of the Pacific Basin Consortium, held at the East–West Center in Honolulu, Hawaii, September 2005 and supported by the National Institute of Environmental Health Sciences (NIEHS) Superfund Basic Research Program, the U.S. Environmental Protection Agency (EPA), and the World Health Organization (WHO). Over most of its 20-year existence, the PBC has focused on regional hazardous waste issues, emphasizing human health effects, regional cooperation, dissemination of information on new technologies, and assistance for training efforts. Recently this focus was broadened to include important regional environmental issues that extend, to a degree, beyond the hazardous waste field. Those additional areas of emphasis are evident in the PBC conference proceedings, which include sessions devoted to air pollution, climate change, and more. It is assured that hazardous materials and related adverse health effects will remain a central theme of the PBC. Industrialization of Pacific Rim nations ensures that the production, release, and disposal of hazardous substances will produce problems that differ in character and scale from those experienced previously. For example, chemical hazards not previously known in parts of the Pacific Rim will become household words, as they have in parts of the world that have already experienced a full measure of industrial development. The character and scale of air pollution problems are likely to change regionally, and water-related problems will become more acute as competing uses for limited water resources arise.

Difficulties related to the breadth of regional challenges to environmental stability and human health among Pacific Rim nations are reflected, albeit more modestly, in the organization of this present report on the 2005 PBC conference. We have elected to describe both the breadth and depth of conference activities. Our approach is to enumerate perceived threats to regional environmental stability and human health, covering briefly specific aspects of those issues that received attention at the conference. Arsenic management, which was featured at the PBC conference, is described in greater depth, taking advantage of continuity in complementary conference presentations in that area.

## Focus of the Conference

Conference sessions were organized around existing or projected threats to sustained growth and environmental quality in the Pacific Basin, with particular focus on human health and hazardous waste. The pace of economic expansion in the Far East, particularly in the fields of industry and manufacturing, is sure to leave a signature on the environment. In China, for example, the management of waste from a relatively new electronics industry, including materials recycling, has resulted in significant exposures of workers and their families, and dwellers proximate to industrial sites to a variety of hazardous chemicals ranging from metals to flame retardants. Polybrominated diphenyl ethers, levels of which have increased exponentially among Europeans and North Americans and whose manufacture and use in those countries has become increasingly restricted, are now the subject of scrutiny by responsible government institutions in China. The developing body of hazardous waste information, which tends to be chemical specific and anecdotal in character, must evolve into a clear focus on the costs of chemical exposure in terms of human and environmental health and then into a management strategy such as has arisen in Europe and the United States. In the following sections, we briefly describe the conference focus area.

### Air quality

Emissions from internal combustion engines and energy-generating facilities are sources of pollution-related health effects in heavily populated, industrialized areas around the entire Pacific Rim. Mexico City and Santiago, Chile, are excellent examples. It is estimated that more than 100 million people are exposed to air pollution levels exceeding WHO standards in Latin America alone. There is clear evidence that cardiovascular and respiratory deaths are correlated to air pollution levels, and pollutants are major triggers for asthma attacks, respiratory infections and bronchitis. A 10% reduction in particulate air pollution in 38 Latin American cities with complete data sets would reduce premature deaths by about 5,000 per year among the 100 million inhabitants. Compliance with the WHO standard for PM_10_ (particulate matter with an an aerodynamic diameter ≤ 10 μm) would lower the premature death rate by 16,000. The economic impacts of air pollution in terms of work days lost and premature deaths are staggering. Restricting the sulfur content of fuel sold in Hong Kong in 1990, for example, reduced local sulfur dioxide levels by 80% and lowered rates of bronchitis among primary school children substantially. Furthermore, related mortality reductions among those in middle life and older were estimated to provide an additional 10,000 person-years to the lives of Hong Kong residents each year. Automobile proliferation and consequent fuel demands are predicted to outweigh recent emphasis on cleaner fuels, however, perhaps negating recent improvements in Hong Kong air quality and attendant health benefits. The estimated cost due to current pollution levels in Hong Kong was estimated at US$2.6 billion per year because of a combination of increased disease incidence and treatment costs, productivity loss, and mortality.

Air pollution costs in terms of human suffering may be greater. The poorest segment of urban society lives in closest proximity to the highest pollutant levels, a social manifestation that is sure to increase with the increasing size of cities in the developing world. More than half the world’s population relies on a variety of fuels consumed indoors without ventilation for routine cooking and eating. Indoor pollution from these practices adds materially to particulate exposure.

There is growing recognition that even remote areas are affected by air pollution. There is clear evidence, for example, that air quality in U.S. national parks, far removed from industrial sources and heavy automobile use, is affected by human activities at distant locations. Transport of air quality problems across national boundaries emphasizes the need for international cooperation in this area. Such cooperation was necessary for the management of acid rain in industrial nations of North America and Europe. The same or greater need is recognized for the management of mercury from atmospheric sources. It has been estimated that 20% of the mercury contamination in North America comes from global sources and that the combustion of coal, incineration, and metallurgical processes are responsible for most of the mercury releases. There is little doubt that these problems will be aggravated through energy demands and growth among major economies in nations of the Pacific Rim.

### Climate change

Discussion of inter-regional and international environmental problems recently has been dominated by anthropogenic influences on global climate. Practical decisions related to management of carbon dioxide emissions have potentially broad economic consequences but uncertain geophysical outcomes. Climate change is of particular relevance among Pacific Rim nations because of the likelihood of sustained regional economic growth and associated dependence on fossil fuel consumption, the concentration of major populations at low elevations, and the fragility of unique Pacific ecologic features. The probable effects of climate change on human and environmental health have not received sufficient attention. Increased disease is likely to result from the spread of vectors of tropical diseases such as malaria, dengue, Chagas’s disease and perhaps others such as the Ebola virus into previously temperate regions of the world. Heat itself is an issue, especially among the elderly and very young, as observed during well-documented heat waves in middle America in 1995 (Semenza et al. 1996) and in Europe in 2003 (Vandentorren et al. 2004). These events allow us to anticipate heat-related health effects in a warming world. Analysis of Japanese meteorologic data from 1972–1995 suggests that *a*) most heat-related deaths are among the elderly; *b*) the incidences of cardiovascular and respiratory diseases are temperature dependent; *c*) the severity of temperature effects on mortality is inversely related to local average temperature (implying that humans may be capable of adapting to climate change, although response time cannot be predicted); and *d*) the frequency of accidents among children is temperature related, in part because of the dependence of activity patterns on temperature. The influences of development, fuel demand, and climate on regional politics are too complex for analysis. Specific aspects of climate-related politics were, however, presented at the PBC conference. The likely role of the recently formed Asia-Pacific Partnership on Clean Development and Climate, for example, both acknowledges the importance of regional economic activity to climate stabilization and provides an avenue for U.S. involvement in climate-related issues outside the Kyoto Protocol, where pressure can be placed on developing countries to curb growth in greenhouse gas emissions. It is well recognized, for example, that China can play a significant role as a supplier of carbon credits that can become available if advanced countries invest in emission controls for Chinese industries.

### Pesticides/herbicides

The health-related effects of pesticide and herbicide use are of equal regional importance and much better defined. Pesticides are a US$32 billion per year industry worldwide, and imports of these products represent, in some cases, 80% of farm-chemical imports in developing countries. The effects of pesticides on humans generally are investigated through a combination of measurements that include direct chemical analysis of blood or other tissues, enzyme (e.g., acetylcholinesterase) activities and genetic observations indicative of DNA damage. Among workers from a flower plantation in Ecuador that routinely employed aerial application methods, the pesticide-exposed group showed significantly higher levels of chromosomal aberrations (~ 16% compared with 3% in the control group) and other indications of genotoxicity than their unexposed counterparts. In general, the assessment of exposure and, consequently, the risk of exposure are extremely complicated because pesticides are routinely used in complex mixtures, frequently at high doses. It has been estimated that from 20 to 200 chemicals are present in some mixtures (Paz-y-Miño et al. 2002). No pesticide has been better studied than dichlorodiphenyl-trichloroethane (DDT) and its metabolites. DDT was used extensively in Mexico for malaria control. Annual application rates decreased from 9,000 to 2,000 tons from 1971 to 1991 because of the suspected effects of DDT on wildlife. Production and use of the chemical were banned in Mexico in 1997. Nevertheless, metabolites of DDT are present in 90% of human samples in Mexico. In 1999, serum levels in women of Morelos (a predominantly agricultural state in southern Mexico) were similar to those of women of agricultural regions of California some 20 years earlier. At least 6% of breastfed children in Mexico City receive unacceptable levels of DDT via this exposure route. An ongoing study suggests that there is an exposure–response relationship between levels of DDT metabolites and impaired motor development in children at 3 months of age (Torres-Sanchez et al. 2007).

### Children’s health

The complexity of developmental processes and their relative sensitivity to chemical exposure led to selection of children’s health as a session topic at the PBC conference. Opportunities for chemical exposures and consequent health problems among children are elevated among developing Pacific Rim nations because of child labor and hazardous waste norms. Labor costs in developed countries have motivated companies to transfer manufacturing operations to developing nations. Although this brings employment to underemployed populations, there is also a net transfer of hazardous waste problems into the developing world. These considerations have nurtured concern for children’s health, particularly among the nations of the Pacific Rim that exhibit the most rapid economic advancement. In Tamil Nadu, a province of southern India, it is estimated that > 2 million children are employed in a variety of cottage manufacturing industries, where they are vulnerable to occupational diseases and injuries. Work in the weaving industry, for example, starts at 7 years of age.

Although environmental pollutants are potential risk factors for both the induction and aggravation of childhood asthma, there are probably multiple contributors to asthma incidence, thereby increasing the difficulty of determining cause and effect. Asthma prevalence in developed nations has increased, for example, during a period of prolonged air quality improvement, and there exist areas of low asthma incidence in heavily polluted Asian megacities. There does appear to be a correlation between asthma incidence and traffic density, however, suggesting that environmental contributors to childhood asthma need to be considered within a context provided by other factors that are genetic and economic in nature.

### Mercury

Papers presented were in general agreement regarding the need to manage mercury in fish and/or the ingestion of fish for management of mercury exposure, although the severity of levels encountered and associated risks differed substantially. Related actions included general guidance or guidelines relative to the ingestion of fish taken from Hawaiian waters in the form of a pamphlet designed for pregnant women, nursing mothers, and young children. On the Tapajos River in the Brazilian Amazon, where mercury release was attributed to large-scale deforestation and slash-and-burn practices, degradation of motor and visual functions among human subjects was correlated with mercury levels in hair and ingestion of certain kinds of fish. Subjects were advised to “eat more fish that don’t eat other fish,” which resulted in declining exposure and recovery of motor skills. An alternative mercury exposure route described was specific to small-scale artesanal gold miners in the Philippines. The miners had blood mercury levels ranging from < 10 to 120 ppb. Health effects included poor memory, anosmia, abnormal gait, and poor balance. On a much larger scale, Feng and co-workers (unpublished data) attempted to account for the rather sizable contribution of mercury to the environment from a variety of activities in Guizhou, a 170,000 km^2^ area of China that contains > 60% of that nation’s mercury resources. Guizhou is also a major coal mining area, yielding coals that are relatively mercury rich. Coal combustion has led to high atmospheric levels of mercury and higher than normal rates of mercury deposition in rainfall.

### Electronic waste

The relative cost of skilled labor has motivated the exodus of many manufacturers to Taiwan, China, Korea, and India. Even high-profile industries such as manufacturers of printed circuit boards that emphasize technical innovation are subject to these realities. The lack of reliable hazardous waste facilities presents a challenge to developing nations, and storage is frequently substituted for treatment and recovery. The increasing dominance of Asian countries in the manufacture of printed circuit boards and computers has changed the landscape of hazardous waste management. Printed circuit manufacture is attended by creation of metal-contaminated wastes and the inevitable computer and information technology scrap. Given the combined advances in software, memory storage, and computer speed, older functional equipment quickly becomes obsolete. Efficiencies in manufacturing have made integrated electronic devices so inexpensive that repair, upgrading, and remanufacture are not economical. Economic forces and the consequent shift in manufacturing emphasis will continue to transfer hazardous waste management challenges into the developing world.

In the United States, from 14 to 20 million personal computers are replaced annually. Only 20–30% are resold. Discarded electronic devices constitute an estimated 2–5% of the nation’s municipal solid waste stream. The European Union (EU) generates about 6 million tons of electronic waste per year containing about 0.65 million tons of copper in addition to a host of other metals and plastic components that are difficult to recover. Toxic chemicals widely used in electronic devices include arsenic, cadmium, chromium, copper, lead, and mercury. Typical circuit boards contain resin, fiberglass, copper, brominated flame retardants, and other organic chemicals. Cathode ray tubes from computer monitors and televisions contain barium, cadmium, copper, lead, zinc, and rare earth metals.

Landfill disposal will remain the dominant disposal method for these materials. The landfill experience in North America in preventing contamination of groundwater by waste sites has not been ideal. The number of waste sites in North America requiring remediation has been growing rather than decreasing, and associated remediation costs are exceptionally high. There is an opportunity for developing nations to learn from the experience of the industrialized world and take a more proactive path. Because wastes almost by definition have little value, the costs for processing, recovery, and reuse will have to be borne by the consumer. Without a take-back fee for recycling, there is almost no possibility for a sustainable waste diversion industry in North America. These costs are already accepted by the public for other items. For example, in Canada a deposit is paid when an automotive battery is purchased, and part of that deposit is refunded when the battery is returned for recycling. The rest of the fee is used to assist the recycling of the battery.

The labor costs in North America make the waste processing/recovery of more integrated products such as computers very difficult, even with subsidies. The two largest costs are for sorting and dismantling. The lower wage scales in the developing world will inevitably motivate the flow of waste computer scrap to these areas. In 1998 Taiwan’s EPA declared scrap computers to be a producer responsibility product, whereby manufacturers and importers of personal computers are responsible for the recovery and recycling of their products. Five recycling plants have been established locally to treat the collected scrap computers.

Unrelated investigations in China underscore the level of exposure experienced by residents living proximate to municipal landfills in those countries. Polychlorinated biphenyls (PCBs) provided the bulk of the data presented. These and other hydrophobic contaminants are biomagnified by factors on the order of 100,000 in the aquatic food chain, approaching parts-per-thousand concentrations in lipid-rich fish tissues. These levels are 10× higher than PCB concentrations that alarmed public health officials when measured in trout from Lake Ontario in the United States in 1996. Hen eggs provide another effective and more-or-less universally available means for establishing the distribution of persistent organic pollutants (POPs) in the vicinity of landfills and for estimating human exposures.

The investigators in China concluded that E-waste (electronic waste) recycling was the major source of POP contamination. Hydrophobic contaminants spread widely due to entrainment and re-disposition of landfill dust, and biomagnification led to unacceptable human exposure via ingestion of food (especially rice seeds, fish and hen eggs). Their measurements indicate that *in utero* exposure to PCBs is a concern. The population living proximate to a landfill that received E-waste experienced much higher exposures and higher body burdens of POPs than those at a rural control location.

The study in Pakistan showed that exposure to POPs via ingestion of free-range chicken eggs was much greater in households nearby landfill sites and incinerators. It was determined that even straightforward, economical hazardous waste management measures could reduce human exposure significantly.

There are now comparative data for New York state populations in ZIP codes with *a*) waste disposal sites that handle POPs, *b*) disposal sites without POP facilities, and *c*) no hazardous waste disposal facilities (Carpenter et al. 2007). After correcting for potentially confounding effects such as age, race, sex, and economic status, it was determined that risk of acute myocardial infarction, stroke, hypertension and diabetes increased in the ZIP codes containing POP disposal facilities. An extension of the same study was carried out in a subset of ZIP codes along the Hudson River in New York to minimize the effects of behavioral factors such as smoking, exercise, and diet. Results were essentially unchanged.

## Managing Arsenic in Potable Waters

Arsenic in drinking water is a problem of sufficient breadth and magnitude among Pacific Rim nations to warrant special consideration by the PBC. Arsenic also exemplifies the degree to which multidisciplinary skills are focused on hazardous waste management issues within the PBC context. Complementary presentations in arsenic toxicology, treatment, and disposal technologies provide a model of technology transfer that the PBC hopes to emulate in other areas. There were individual papers on *a*) arsenic exposure routes and arsenic health effects; *b*) principles governing the effectiveness of water treatment methods for arsenic removal; *c*) disposal of arsenic-bearing residuals (spent sorptive media) from water treatment; and *d*) the link between arsenic exposure and coronary heart disease. Human health effects have been attributed to arsenic in potable water in Taiwan, Mongolia, Chile, China, India, and Bangladesh. Arsenic-related illness in Bangladesh and West Bengal was called the worst public health calamity in 50 years. Ironically, the severity of arsenic-related illnesses in Bangladesh was a product of western intervention in regional water supply that was designed to protect against water-borne disease (Nickson et al. 1998).

Chronic exposure to inorganic arsenic levels on the order of 300 μg/L or higher in drinking water produces excess cancers of the skin and several internal organs, especially the lungs and bladder, in humans. For perspective, in the U.S. dietary intake of inorganic arsenic is 8–15 μg/day. *In utero* exposure of mice to inorganic arsenic leads to excess liver cancer incidence in adult life. Arsenic exposure contributes to various vascular disturbances (e.g., blackfoot disease), increased risk for diabetes, and others, perhaps at much lower levels in water. It has also been suggested that the risk of arsenic exposure during development is greater than later in life and that children metabolize arsenic differently than adults.

**Figure 1:**
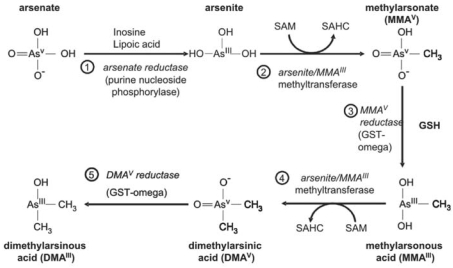
Known pathways of arsenic metabolism in vertebrates. Abbreviations: GSH, glutathione; GST-omega, glutathione *S*-transferase omega; SAHC, *S*-adenosylhomocysteine; SAM, *S*-adenosylmethionine. Note the need to consider six arsenic forms when exposure may be to arsenate alone. Reproduced from Aposhian et al. (2004) with permission from Elsevier.

Arsenic metabolism and its relationship to disease incidence are important areas of current study. Inorganic arsenic and its four known methylated forms are all candidate carcinogens until more information is gathered (Figure 1).

Very large population differences in excreted arsenic forms indicate that there may be important genetic differences in human patterns of arsenic metabolism. The examination of genes that are directly involved in these transformations is an area of exceptional promise. Furthermore, the use of modern analytical methods (e.g., high performance liquid chromatography–inductively coupled plasma–mass spectrometry) and biochemical techniques (e.g., proteomic analysis of arsenic-induced protein synthesis) promises to increase the pace of arsenic-related health effects research. Initial comparisons of liver protein profiles in mice treated with arsenic at levels that produce acute toxic effects indicate that exposure produces rapid (4-hr) up-regulation of genes involved in respiration (electron transport chain components), antioxidant response, and protein folding.

By far the most important step in arsenic-related interventions for protection of human health is interruption of the primary route of exposure. If this can be accomplished, there are chelating agents that show promise as reagents for rapid arsenic dispersal and excretion. These include DMPS and DMSA (2,3-dimercapto-1-propane sulfonate and meso-2, 3-dimercaptosuccinic acid, respectively).

**Figure 2:**
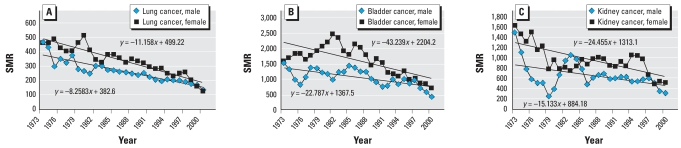
Trends in arsenic-related cancer mortalities after mitigation of arsenic levels within the Taiwan study area. SMR, standardized mortality ratio. Lung cancer (*A*), bladder cancer (*B*), and kidney cancer (*C*) mortalities since 1973 are illustrated. Trend lines from simple linear regression analyses are shown. Modified from Yang et al. (2005) and Chiu et al. (2004).

The data on which the U.S. drinking water arsenic standard was premised were developed from well-water concentrations and cancer rates in Taiwan (U.S. EPA 2001). Among the uncertainties in the benefit/cost comparison developed by the U.S. EPA to support their 2001 regulation was the lag between water quality improvements mandated by the rule and consequent improvements in the public health. While delay between implementation of the new rule and improved health was anticipated, the lag time was impossible to anticipate. Consequently, the U.S. EPA selected a zero-lag model in their analysis and may have overestimated the benefits attributable to the revised maximum contaminant limit for arsenic. Many of the Taiwanese communities that contributed to the epidemiologic database no longer used arsenic-contaminated water for drinking and cooking after the mid-1970s. Drinking water concentrations of arsenic were 350–1,140 μg/L before the change in water and < 10 μg/L afterward. Because records of disease incidence are continuous to present, there is now a database with which to approach the issue of lag time between lower arsenic exposure and improved health. Mortalities from lung, bladder, and kidney cancers declined continuously since 1973 among both men and women in the affected Taiwanese communities (Figure 2). The record (1973–2000) suggests that cancer mortality rates may not have reached background levels by 2000, however. Half-times for reestablishment of normal cancer rates were at least 15 years in length (Chiou et al. 2001).

Because the incidence of coronary heart disease (CHD) among men decreased substantially in the same study area over the same period, a causal connection between arsenic ingestion and male CHD was inferred. A similar trend among women was not apparent. This is perhaps the best epidemiologic data with which to assess the lag between arsenic exposure and recovery from arsenic-related disease incidence in humans.

Cost-effective methods for removing arsenic from water are based on adsorption or ion exchange. The propensity of inorganic arsenic to partition on solids depends on arsenic oxidation state, solution pH, and physical–chemical characteristics of the solid medium. As(V) is the preferred oxidation state for removal on solid material (U.S. EPA 2001). Arsenate is negatively charged in the pH range of most natural waters. Solid media designed for arsenic removal are generally aluminum- or iron-based to take advantage of their ability to chemisorb arsenic in the neutral pH range. An extensive database indicates that freshly precipitated amorphous granular ferric hydroxide (AFH) offers advantages over most alternative media for removal of arsenic from water via adsorption.

Among the primary advantages of AFH for anion adsorption is the very large specific surface area of the freshly precipitated mineral. The material, however, is in a state of continuous physical–chemical flux, as the poorly consolidated amorphous mineral that is formed initially evolves toward a more thermodynamically favored crystalline structure. Thus surface area declines significantly with mineral age, leading to reduction in sorptive capacity and loss of previously adsorbed arsenic. A few innovative solutions are available to avoid these limitations, including the strategy outlined here.

J. Farrell and D. Mishra (unpublished data) used mixed valent oxides of iron and manganese as reactive adsorbent media for removal of arsenic from water. Corrosion and dissolution of the media in oxygenated waters continuously generates Fe(III) and Mn(IV) species that remove arsenic via coprecipitation and adsorption onto freshly precipitated Fe(III)/Mn(IV) hydroxides. Column experiments were performed to determine the effectiveness of the media for arsenic removal over a range in empty bed contact times, influent arsenic concentrations, dissolved oxygen levels, solution pH values, and silica concentrations. Corrosion and dissolution of the media were sufficiently slow to avoid column clogging over 90 days of operation. The reaction products formed in the columns contained average As-to-Fe ratios that were more than 4 times higher than those achieved by commercially available granular ferric hydroxide media under similar conditions. The higher arsenic loadings are likely due to a coprecipitation mechanism and the fact that freshly precipitated ferric hydroxides have higher arsenic adsorption capacities than aged materials. The new adsorbent media can be used in packed-bed filters in both large- and small-scale treatment systems as well as point-of-use filters suitable for individual wells or faucets. No regeneration of the adsorbent media is planned.

The magnitude of arsenic-related water quality problems in the United States alone is likely to yield an estimated 6.0 × 10^6^ pounds of spent sorptive media (mostly iron-based sorbents saturated with arsenic) per year (Ghosh et al. 2006a). Safe disposal in sanitary landfills relies on an assumption that spent media will not be considered a hazardous waste within rules pursuant to the Resource Conservation and Recovery Act of 1976 (1976). For such determinations, the U.S. EPA relies on an operational test called the Toxicity Characteristic Leaching Procedure (TCLP). The TCLP measures the extent to which chemicals can be resolubilized from a solid waste under specified (acidic conditions). Should the test yield a liquid-phase arsenic concentration ≥ 5 mg/L under TCLP conditions, the spent sorbent will be a hazardous waste requiring special handling, transport and disposal methods (U.S. EPA 2001).

The relevance of the TCLP for arsenic leaching tests was brought into question by data that compare arsenic leaching rates in alternative test methods, including methods that are designed to simulate chemical and biochemical conditions in sanitary landfills (Ghosh et al. 2006b). The alternative tests produced much higher levels of arsenic in leachates resulting from granular ferric hydroxide (GFH) and alternative sorbents for arsenic removal from water. The major flaws in the TCLP procedure for arsenic leaching are the use of a low-pH solvent to solubilize an anionic sorbate (high pH solutions would be more aggressive); maintenance of aerobic conditions during the test; and absence of microbial activity, as microbes could greatly enhance the rate of sorbent breakdown and arsenic release. Tests in a column simulation of landfill conditions indicated that although leachate arsenic levels never reached 5 mg/L in the continuous flow apparatus, more than 50% of the arsenic initially present in GFH added to the column was solubilized over a period of 1,000 days. GFH was reduced to Fe(II) and leached from the column reactor at a similar rate until the iron was essentially gone (Ghosh et al. 2006b), after which the rate of arsenic loss from the column increased substantially.

**Figure 3:**
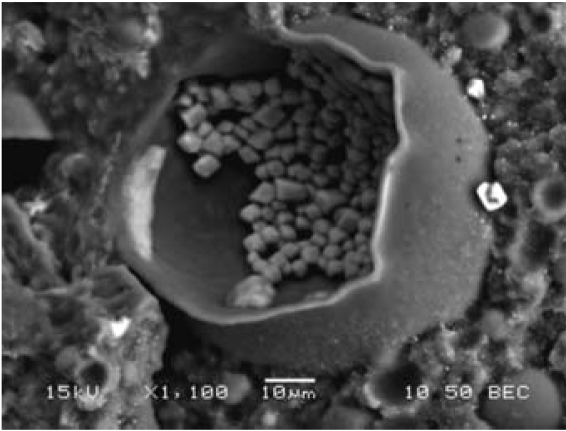
A scanning electron image of sodium nitrate encapsulated in polymeric matrix. Salt crystals form inside polymeric “sacs” leading to stable encapsulation. Reprinted from Rengifo et al. (2004) with permission from the American Chemical Society.

Candidate technologies for arsenic immobilization include vitrification and encapsulation in cement or a more suitable alternative material. Vitrification is highly energy intensive and was not seriously considered. Cement encapsulation, although inexpensive, permitted rapid loss of arsenic when the encapsulated product was immersed in aqueous solutions, similar to leachates in mature landfills. A polymeric waste encapsulation strategy proved much more suitable. The polymeric form was produced from polystyrene butadiene rubber and epoxy resin. The residuals were dispersed in an emulsion of polymer precursors. Drying and curing yielded a solid waste form that was mechanically durable, chemically stable, and relatively inexpensive (Ela et al. 2006). Initial experiments with a soluble salt (sodium nitrate) showed an effective encapsulation of the solid at the microscopic level (Figure 3) with a higher degree of stability than cement encapsulation. Sodium nitrate was leached from a cement-encapsulating form more than an order of magnitude faster than from the polymeric matrix under the same leaching conditions. Subsequent tests showed that Asladen GFH is a suitable substrate for polymer encapsulation, with stable waste forms containing as much as 65% sorbent by weight.

The breadth of arsenic-related talks—from waste generation/risk analysis, through treatment, and including residuals disposal—illustrates the interdisciplinary approach to hazardous waste management that is possible through the PBC. The twelfth PBC conference was held 26–29 October 2007 in Beijing, China.
